# Rationale in diagnosis and screening of atrophic gastritis with stomach-specific plasma biomarkers

**DOI:** 10.3109/00365521.2011.645501

**Published:** 2012-01-12

**Authors:** Lars Agréus, Ernst J Kuipers, Limas Kupcinskas, Peter Malfertheiner, Francesco Di Mario, Marcis Leja, Varocha Mahachai, Niv Yaron, Martijn Van Oijen, Guillermo Perez Perez, Massimo Rugge, Jukka Ronkainen, Mikko Salaspuro, Pentti Sipponen, Kentaro Sugano, Joseph Sung

**Affiliations:** 1 Karolinska Institute, Center for Family and Community Medicine, Stockholm, Sweden; 2 Department of Gastroenterology and Hepatology, Erasmus MC University Medical Center, Rotterdam, The Netherlands; 3 Institute for Digestive Research, Lithuanian University of Health Sciences, Kaunas, Lithuania; 4 University, Department of Gastroenterology, Hepatology and Infectious Diseases, Magdeburg, Germany; 5 Department of Clinical Sciences, University of Parma, Section of Gastroenterology, Parma, Italy; 6 Riga East University Hospital, Digestive Diseases Centre, Riga, Latvia; 7 Department of Medicine, Division of Gastroenterology, Chulalongkorn University, Thailand; 8 Department of Gastroenterology, Rabin Medical Center, Petah Tikva, Israel; 9 Dept. Gastroenterology and Hepatology, University Medical Center Utrecht, Utrecht, The Netherlands; 10 WAMC, New York, USA; 11 Department of Pathology, University of Padova, Padova, Italy; 12 Primary Health Care Center, Tornio, Finland; 13 University of Helsinki, Research Unit on Acetaldehyde and Cancer, Helsinki, Finland; 14 Patolab Oy, Espoo, Finland; 15 Department of Internal Medicine, Division of Gastroenterology, Jichi Medical University, Tochigi, Japan; 16 Institute of Digestive Disease, Prince of Wales Hospital, The Chinese University of Hong Kong, Hong Kong, China

**Keywords:** acetaldehyde, achlorhydria, atrophic gastritis, biomarker, calcium, gastric cancer, gastrin, Helicobacter pylori, pepsinogen, vitamin B_12_

## Abstract

**Background and aims:**

Atrophic gastritis (AG) results most often from *Helicobacter pylori (H. pylori)* infection. AG is the most important single risk condition for gastric cancer that often leads to an acid-free or hypochlorhydric stomach. In the present paper, we suggest a rationale for noninvasive screening of AG with stomach-specific biomarkers.

**Methods:**

The paper summarizes a set of data on application of the biomarkers and describes how the test results could be interpreted in practice.

**Results:**

In AG of the gastric corpus and fundus, the plasma levels of pepsinogen I and/or the pepsinogen I/pepsinogen II ratio are always low. The fasting level of gastrin-17 is high in AG limited to the corpus and fundus, but low or non-elevated if the AG occurs in both antrum and corpus. A low fasting level of G-17 is a sign of antral AG or indicates high intragastric acidity. Differentiation between antral AG and high intragastric acidity can be done by assaying the plasma G-17 before and after protein stimulation, or before and after administration of the proton pump inhibitors (PPI). Amidated G-17 will rise if the antral mucosa is normal in structure. *H. pylori* antibodies are a reliable indicator of helicobacter infection, even in patients with AG and hypochlorhydria.

**Conclusions:**

Stomach-specific biomarkers provide information about the stomach health and about the function of stomach mucosa and are a noninvasive tool for diagnosis and screening of AG and acid-free stomach.

## Introduction

An expert group appointed by IARC (International Agency for Research on Cancer) concluded in 1994 that the *Helicobacter pylori (H. pylori)* infection is a group I carcinogen to humans [1]. However, the mechanism by which the *H. pylori* infection causes noncardia (distal) gastric cancer has remained obscure. *H. pylori* infection induces chronic active gastritis that develops with time in a proportion of infected people to atrophic gastritis (AG) and acid-free or hypochlorhydric stomach [2,3]. In AG, focal neoplastic (dysplastic) lesions may appear that gradually progress into an invasive malignancy. On average, this so-called Correa cascade concerns approximately one-half of the gastric cancer cases [4]. Early treatment of the infection is considered an action of choice, as it may slow or intercept the Correa cascade [5,6].

AG of the stomach mucosa is the highest known independent risk factor (risk condition) for distal, noncardia gastric cancer [4,7,8,9,10]. By definition, atrophy means a loss of normal antral and/ or oxyntic glands. This loss is accompanied by fibrosis of the *lamina propria* and by the appearance of new metaplastic glands of intestinal and/or pseudopyloric type in the injured mucosa. AG progresses slowly and may finally result in severe, advanced atrophy, i.e., in total or nearly total loss of normal mucosal glands [3,11,12]. Curable precancerous lesions and early cancers are frequently found in stomachs with severe AG and intestinal metaplasia. In an endoscopic study from Finland, a definite neoplastic lesion was found in 63 (4.7%) of 1344 men (age 50–69 years) with a low plasma level of pepsinogen I (PGI), and with moderate or severe corpus AG in the endoscopic histology. Of these 63 lesions, invasive cancer was found in 11 cases (in 7 patients the cancer was in an “early” stage, i.e., invasion limited to the submucosa). High-grade intraepithelial neoplasia (dysplasia) was found in 7 men, low-grade intraepithelial neoplasia in 42 men, and an ECL cell type carcinoid tumor in 3 men [10].

Cancers appear in patients with nonatrophic *H. pylori* gastritis as well, but are more infrequent than that in AG [6,13]. Eradication of *H. pylori* early enough is considered the key to preventing distal stomach cancer, provided that the presence of neoplastic or preneoplastic lesions, AG, or intestinal metaplasia is excluded before the treatment [13,14]. It has recently been estimated in China that one treatment of *H. pylori* might prevent one distal gastric cancer in every four to six cases undergoing the *H. pylori* eradication [15].

On average, half of the case with *H. pylori* infection will develop AG of some degree during their lifetime, and in around 10% of the infected subjects, the AG will finally be moderate or severe [3,16,17]. In the latter category, 2.5–5% may get a cancer [10].

In *H. pylori* infection, gastritis (chronic mononuclear inflammation) and atrophy (loss of normal mucosal glands) tend to appear first in the antrum and angulus and will tend to progress by pylorocardial extension [11]. The “atrophic border,” which can even be seen in ordinary endoscopy, moves upward with time, finally resulting in AG that occupies the whole stomach [11].

*N*onatrophic *H. pylori* gastritis raises the risk of gastric cancer fourfold on average, and the risk may rise to 15-fold in patients with AG [13]. In subjects with severe panatrophy (AG in both antrum and corpus, i.e., severe multifocal atrophic gastritis), irrespective of the presence or absence of ongoing *H. pylori* infection, the cancer risk may even be up to 90-fold compared with the risk in subjects with a healthy stomach mucosa [8].

Eradication of *H. pylori* will inevitably improve stomach health in subjects with nonatrophic *H. pylori* gastritis, or even with mild gastric atrophy as indicated, for example, by an increase in the serum levels of PGs after a successful *H. pylori* therapy [18]. Severe precancerous conditions or lesions, like AG, intestinal metaplasia or dysplasia, may not always regress and may even progress to invasive cancer despite a successful *H. pylori* eradication [13]. This can even occur at intervals longer than a decade [14,19].

The plasma biomarker test can be used for the screening of patients with a “sick stomach mucosa” and for those with AG in particular, i.e., patients eligible for gastroscopy and endoscopic surveillance for cancer risk [20,21]. The biomarker screening would help, in addition, in the identification of the patients with a “healthy” stomach mucosa, in whom the cancer risk is low, and in whom the endoscopy may not be the first important diagnostic procedure.

In a recent survey with a biomarker panel among 4256 Finnish adult volunteers (mean age of 56 years; range 18–92 years), the overall prevalence *of H. pylori* infection was 19% in the whole population studied, and the prevalence of moderate or severe (advanced) atrophic corpus gastritis was 6% (110 persons) among people aged 60 or more [22]. Since the cancer risk in advanced AG is around 5%, one may estimate that at least 6 people out of the 4256 persons screened would be liable to develop stomach cancer. It is especially noteworthy that the stomach mucosa could be classified as normal and healthy (no *H. pylori* gastritis, no AG) by the biomarkers in 77% of all 4256 subjects analyzed [22].

Cancer risk is not the only medical challenge associated with an achlorhydric stomach and AG. AG and acid-free stomach may also lead to other diseases than the gastric illnesses alone. Such “extra-gastric” diseases are, for example, malabsorptions of vitamin B_12_ and malabsorption of certain micronutrients and Pharmaceuticals, or an increased risk of gastrointestinal (GI) and pulmonary infections, especially among the elderly [23,24]. From viewpoints of the public health, these non-neoplastic and extra-gastric consequences of AG and acid-free stomach may even be more important than the cancer burden.

## Objectives of the paper

The present paper is a summary of a set of studies published on the application of stomach-specific biomarkers in noninvasive diagnosis of AG. We focus on reviewing the applicability, background, and rationale of the PGI and PGII, gastrin-17, and *H. pylori* antibodies in assessment of the stomach health, and in screening of the AG and acid-free stomach. Several excellent reviews on the use of PGs alone have been published earlier [25,26,27,28]. The present paper focuses on the application of a more comprehensive set of tests in which also the plasma levels of PGII, amidated gastrin-17, and *H. pylori* antibodies are noted and assayed, in addition to the PGI alone. We argue why the screening and diagnosis of AG with biomarkers are noteworthy in clinical practice, and we suggest how the interpretation of the biomarker tests could be carried out.

## The effects of AG on gastric physiology and plasma biomarkers

In AG, the normal functional cells and glands in the gastric mucosa decrease in number and finally totally disappear. In corpus AG, the acid-producing parietal cells and the PG-secreting chief cells will disappear and, concomitantly, the secretion of stomach acid, PGs (pepsins), and intrinsic factor will decrease. The stomach becomes hypochlorhydric and finally achlorhydric (acid free). The decrease in acid output and the decrease in the plasma levels of PGI and the PGI/II ratio correlate well with the grade and extent of the corpus AG, as validated by the biopsy histology or by the pentagastrin test [29,30]. There is some evidence that the PGI/II ratio is a more reliable biomarker for the corpus AG than the PGI test alone, particularly in studies from Asia [20,25]. Atrophy of antral (pyloric) glands results in loss of antral G cells, and, subsequently, in decreased capacity of the antrum to synthesize and secrete amidated gastrin-17 into the circulation.

**Figure 1:**
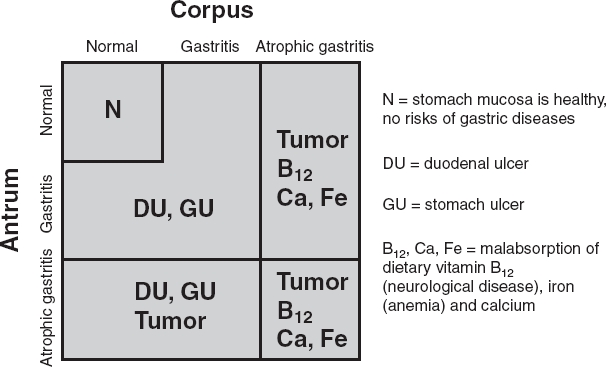
Algorithm on how nonatrophic *Helicobacter pylori* gastritis and atrophic gastritis in different parts of the stomach are linked with risk of gastric cancer, peptic ulcer disease, and with failures in absorption of dietary vitamin B_12_ and some essential micronutrients. Abbreviations: N = stomach mucosa is healthy, no risks of gastric diseases; DU = duodenal ulcer; GU = stomach ulcer; B_12_, Ca, Fe = malabsorption of dietary vitamin B_12_ (neurological disease), iron (anemia), and calcium.

**Figure 2:**
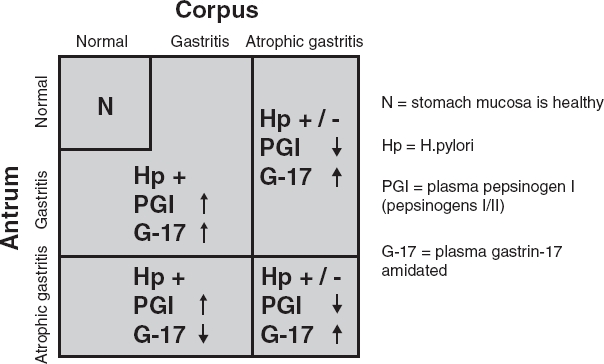
Algorithm on how the plasma levels of stomach biomarkers are linked with nonatrophic *Helicobacter pylori* gastritis (previously so-called superficial gastritis) and with atrophic gastritis of various topographic phenotypes. Abbreviations: N = stomach mucosa is healthy; Hp = *H. pylori*; PGI = plasma pepsinogen I (pepsinogens I/II); G-17 = plasma gastrin-17 amidated.

Parallel assays of PGI, of the PGI/II ratio, and of amidated gastrin-17 comprise an exact and validated set or panel of biomarkers that reflect the degree of mucosal inflammation, the extent and grade of AG in the stomach, and the capacity of the existing mucosa to secrete acid and gastrin-17 [29,31,32]. Thus, the changes in plasma levels of the biomarkers reflect changes in the structure and function of the gastric mucosa, i.e., the abnormal levels are signs of a “sick” stomach mucosa and indicate failures in the feedback mechanism that controls the acid output in the stomach (Figures 1 and 2). Reciprocally, normal plasma levels of these biomarkers indicate that the stomach mucosa is healthy with normal structure and function.

In atrophy of the antral mucosa, and with a concomitant disappearance of the gastrin-synthesizing and secreting G cells, the plasma level of gastrin-17 is low not only in fasting conditions but also in stimulated conditions, i.e., after a protein intake, bombesin (gastrin-releasing peptide) stimulation, or after PPI administration [30,31,32,33]. Conversely, in subjects with a normal number of antral G cells, the plasma level of gastrin-17 will increase markedly after the stimulation. The fasting levels of amidated gastrin-17 tend to be low (<1–2 pmol/l) in subjects at risk for erosive esophagitis and Barrett's esophagus, in cases where the patient suffers from gastroesophageal reflux [34].

Recently, an international group of gastroenterologists and pathologists developed a staging system (OLGA staging) for reporting AG in endoscopic biopsies from antrum and corpus [35,36]. The OLGA system (Operative Link for Gastritis Assessment) categorizes patients into five stages (0–IV) according to the grade and extension of histologically assessed AG in the stomach. In a multicenter follow-up study on OLGA, stages III and IV were significantly associated with an increased gastric cancer risk [34]. At the same time, the staging correlated well with the plasma levels of the stomach biomarkers. The ratio of PGI/PGII decreased with increasing OLGA stage (from 0 to IV).

## Impaired absorption of vitamin B_12_, micronutrients, and Pharmaceuticals in acid-free stomachs

As AG progresses in the gastric corpus and fundus, the secretion of intrinsic factor from oxyntic glands decreases and will finally cease completely. Hypochlorhydria impairs the release of the protein-bound dietary vitamin B_12_, and the lack of intrinsic factor prevents the adequate absorption of the dietary vitamin B_12_ in the small intestine. All subjects with moderate or severe corpus atrophy are at risk for this malabsorption, and thus at risk for neurological and metabolic consequences known to be related to the vitamin B_12_ deficiency [23,37,38].

In a Finnish population-based study, every second patient with moderate or severe corpus AG had, at the time of the diagnosis, a low (170–220 pmol/l) or very low (below 170 pmol/l) serum vitamin B_12_ level, often with a simultaneous increase in the serum homocysteine (over 15 micromol/l) [23]. At present, by extrapolation, up to 8% (15,000) of Finns over the age of 60 belong to this risk category for vitamin B_12_ malabsorption. In Russia there would then be about 500,000 people having severe corpus AG and vitamin B_12_ malabsorption, most of these without proper diagnosis or substitution therapy. In Spain, Germany, and Italy, the corresponding numbers would range from 100,000 to 200,000. In the USA, it has been estimated earlier that approximately 800,000 elderly people have undiagnosed and untreated pernicious anemia due to vitamin B_12_ deficiency [35].

With a diminished gastric acid secretion, the absorption of certain divalent micronutrients such as iron, calcium, magnesium, and zinc may be impaired [24,39,40,41,42]. The release and conversion of the micronutrients into actively absorbable ions require the presence of stomach acid [24]. One may assume that in a significant proportion of elderly people, cognitive disturbances, neurodegenerative and vascular disorders, encephalopathies, anemias, and osteoporosis may be caused by undiagnosed malabsorption of the essential micronutrients and vitamin B_12_ due to a hypochlorhydric or acid-free stomach, and AG [42].

The influence of achlorhydria on absorption of perorally administered pharmaceuticals is poorly known. However, many pharmaceuticals need the presence of stomach acid for proper absorption. For example, the absorption of calcium carbonate, dipyridamole, some iron formulations, and antifungal medicines, such as fluconazole and itraconazole, thyroxin, and atazanavir, are known to be impaired in acid-free subjects [43,44,45,46].

## Risk of GI and pulmonary infections when the stomach is acid free

Stomach acid is a natural defense against oral microbes [47]. An acid-free stomach is not defended, independently of whether the achlorhydria is caused by AG or acid inhibitors (PPI). The risks of pneumonia and intestinal infections (e.g., giardiasis, malaria, *Clostridium difficile,* etc.) have been reported to be increased in hypochlorhydric subjects [47,48,49,50].

## Appearance of carcinogens in an acid-free stomach

An atrophic and achlorhydric stomach is always colonized to a massive extent with bacteria and fungi representing the normal oral flora [51,52,53]. This colonization leads to reduction of nitrates to nitrites, and to formation of potential carcinogenic *N*-nitroso compounds and acetaldehyde in the stomach [4,54,55].

Acetaldehyde is an abundant novel carcinogenic compound in the upper GI tract, in the stomach in particular. In 2009, IARC concluded that the acetaldehyde derived from alcoholic beverages, and formed from ethanol endogenously, is a group 1 carcinogen in humans. This conclusion was based on uniform epidemiological, genetic, biochemical, and microbiological evidence from studies with alcohol-consuming individuals carrying, for example, the alcohol dehydrogenase (ADH) and aldehyde dehydrogenase (ALDH2) gene mutations. In the presence of ethanol, these mutations lead to an increased exposure of the upper digestive tract mucosa to acetaldehyde [52,53,56], resulting in an increased cancer risk [57,58,59,60,61,62,63,64].

Tobacco smoking, which is a significant source of acetaldehyde in the upper GI tract, is a known independent risk factor for stomach cancer [65,66,67,68,69,70]. In a prospective Japanese follow-up study, the risk of stomach cancer was, in comparison with the *H. pylori-negative* nonsmokers, 11-fold among the *H*. pylori-positive smokers, 6-fold among *H. pylori-negative* smokers, and 7-fold among *H*. pylori-positive nonsmokers [69]. As a water-soluble agent, acetaldehyde readily dissolves in saliva and gastric juice in mutagenic concentrations [65]. The esophagus may, in addition, be exposed to acetaldehyde from gastric juice via gastroesophageal reflux. Novel L-cysteine-releasing compounds that are able to bind and inactivate carcinogenic acetaldehyde locally in the stomach may provide a new therapeutic tool to eliminate the acetaldehyde from the stomach [71].

## Why is the diagnosis of AG important in cancer prevention?

Early endoscopic diagnosis of gastric cancer or precancerous lesions (intragastric neoplasia, dysplasia) followed by proper endoscopic or surgical therapy is the best available guarantee to improve the stomach cancer prognosis. The noninvasive diagnosis of precancerous conditions *(H. pylori* gastritis or AG) provides a tool for identification of the subjects at cancer risk, i.e., identification of the subjects in whom a diagnostic endoscopy and cancer surveillance are necessary irrespective of the presence or absence of symptoms.

About one million new cases of stomach cancer appeared in 2008 (988,000 cases, 7.8% of all cancers) worldwide, making the gastric cancer currently the fourth most common malignancy in the world [72]. Because of dismal prognosis, stomach cancer is the second leading cause of cancer deaths in both sexes worldwide (736,000 deaths, 9.7% of total). The highest mortality rates are in Eastern Asia (28.1 per 100,000 in men and 13.0 per 100,000 in women) and the lowest in Northern America (2.8 and 1.5 respectively).

## Cancer risk groups

The risk of stomach cancer is insignificant, nil in practice, in people with a normal, healthy gastric mucosa (no *H. pylori* infection or AG). Exceptions are the rare cases associated with inherited gene errors or specific cancer syndromes [73,74,75,76]. In cases with inherited gene errors, the cancer is usually seen in successive generations, appears often before age 60, and is often of a diffuse subtype [75]. In most countries, even in those with relatively low *H. pylori* prevalence, less than 10% of the gastric cancer patients have a normal gastric mucosa (see Table I).

**Table I:** Age-group specific prevalences of *Helicobacter pylori* nonatrophic (superficial) and atrophic gastritis in patients with advanced (invasive) gastric cancer. The series was collected in Finland in 1980–2000 from consecutive patients referred to endoscopy and/or surgery in a Helsinki university hospital, Jorvi Hospital, Espoo. “Healthy” stomach mucosa means that the patient has no gastritis, no *Helicobacter* infection, nor metaplastic or atrophic changes (AG) in antrum or corpus mucosa in the available surgical and/or endoscopic tissue specimens.

		"Healthy stomach” mucosa	Nonatrophic gastritis	Atrophic gastritis
				
Age group, years	Number of patients	No. (%)	No. (%)	No. (%)
<50	34	5 (15%)	28 (82%)	1 (3%)
50–59	33	3 (9%)	15 (45%)	15 (45%)
60–69	57	6 (11%)	19 (33%)	32 (56%)
70–79	60	1 (2%)	10 (17%)	49 (82%)
80-	22	1 (5%)	2 (9%)	19 (86%)
Total	206	16 (8%)	74 (36%)	116 (56%)

Autoimmunity is one of the etiopathogenetic mechanisms of corpus AG, and patients with autoimmune diseases are, therefore, a special risk group for stomach cancer. On the other hand, AG may be rarely linked to autoimmunity alone. Studies from Italy and Finland indicate that the *H. pylori* infection is the most important cause of AG in Europe, also in the cases in which the AG is limited to the gastric corpus, and in which the phenotype of AG resembles that of the autoimmune disease [77,78,79]. In these studies, signs of an active or a past *H. pylori* infection could be found in 70–80% of the people with advanced corpus AG [79].

The importance of *H. pylori* infection in stomach cancer pathogenesis has also been challenged. According to the so-called Indian/African enigma, gastric cancer incidence is low in some parts of Asia and Africa in spite of a high *H. pylori* infection rate [80,81]. So far, this enigma has remained unexplained.

### Gene polymorphisms affecting acetaldehyde metabolism

Functional gene polymorphism resulting in a deficient ability to detoxify carcinogenic acetaldehyde characterizes some specific groups of people at risk for gastric cancer. A relative risk of 3.5 for stomach cancer has been reported among ALDH2-deficient Japanese heavy drinkers [82,83]. In a more recent Japanese study including 45 alcoholics with gastric cancer and 281 controls, the odds ratio (OR) for those with severe corpus AG in combination with ALDH2 deficiency was 39 as compared with an OR of 18 for those with AG alone and OR of 10 for those with the ALDH2 deficiency alone [60]. The Asian-type ALDH2 mutation is almost nonexistent in Europe. However, in a European multi-center case-control study including 811 cases and 1083 controls, an ALDH2 variant with a deficient ability to detoxify acetaldehyde was found to be associated with a 1.8-fold risk of upper aerodigestive tract cancers among moderate drinkers [84]. The OR was 6 among heavy drinkers. In a study from Poland, the same ALDH2 variant was found to be associated with a 2.3-fold risk of stomach cancer among daily drinkers and 3-fold risk was reported among those with 40 or more drink-years [85].

### Age and sex

The incidence of gastric cancer increases exponentially with age. In multivariate analyses, age is, however, not an independent risk factor for gastric cancer – it is only a surrogate marker [8]. The cancer risk is a result of the prevalence of *H. pylori* gastritis and AG in the cohort of people under examination, not of the age of the people in the cohort. In practice, the gastric cancer risk in a 70-year-old subject with a normal and healthy stomach mucosa (no *H. pylori* gastritis or AG) is as low as the cancer risk in a 30-year-old person with a healthy stomach (see Table I).

Worldwide, the age-specific incidence of gastric cancer of intestinal type is approximately twice as high in males as in females. There is evidence suggesting that this difference is caused by estrogens that protect the females from stomach cancer instead of being a result of any differences in gastritis or AG between the sexes [86].

## Biomarker tests in assessing the stomach health

**Figure 3:**
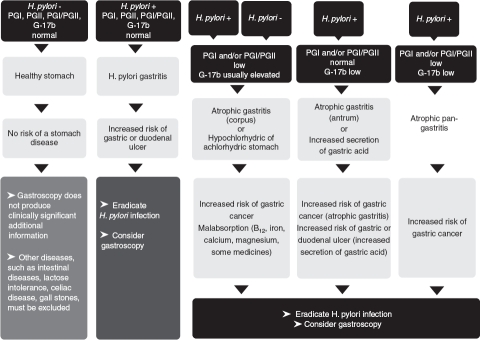
A guide and proposal for the application of a gastric biomarker panel in clinical practice and decision making. Abbreviations: G-17b = plasma level of fasting amidated gastrin-17; Atrophic pangastritis = AG that affects both antrum and corpus (multifocal, advanced, and extensive AG); PGI and PGII = plasma level of pepsinogen I and pepsinogen II, respectively.

Patients with alarming symptoms (bleeding, black stools, weight loss, sudden stomach pains, obstruction, etc.) are to be referred to gastroscopy and to consultation by a gastroenterologist without any prior testing, and even without any prior *H. pylori* tests. In other instances, the estimation of stomach health and the assessment of a need of prompt diagnostic endoscopy can be done easily with the biomarker examination (Figures 1,2,3).

As simple guidelines, the biomarker examinations can be described as follows [83]:

A. *No H. pylori infection, no AG. Stomach mucosa is normal and healthy*. All biomarker tests are normal: Gastroscopy is unlikely to reveal any relevant pathology in the stomach, except on some specific grounds, such as prolonged NSAID use. The normal test results indicate that the stomach mucosa operates normally and the mucosa has normal structure. The risk of gastric cancer or peptic ulcer disease is very small, provided that a patient does not use anti-inflammatory drugs, does not smoke, and has no family history of gastric cancer.
B. *H. pylori infection without AG* (chronic non-AG); only *H. pylori* antibody test is positive, and other biomarker test results are normal. In these cases, the need of gastroscopy is to be decided by the physician and based on the patient's wishes. The risk of gastric cancer is small but not nil. Particularly, diffuse-type gastric cancer can occur in non-AG. All non-neoplastic disease conditions (peptic ulcer disease) heal, and the risk of the stomach diseases decreases if the *H. pylori* infection is eradicated.
C. *AG* (plasma levels of the biomarkers indicate AG, independently of whether the *H. pylori* test is positive or negative): Stomach is acid free (hypochlorhydric/achlorhydric) if AG is in the corpus and is moderate or severe. Cancer risk is noteworthy but the risk of peptic ulcer is nil. Consultation by a gastroenterologist is necessary and a gastroscopy needs to be performed due to increased cancer risk. In patients with a positive *H. pylori* result, eradication of the bacterium should be carried out. In cases with acid-free stomach (severe corpus atrophy), the use of PPIs or other medications for acid inhibition are irrelevant. In subjects with gastroesophageal reflux, the refluxate is not acid if the AG occurs in the corpus and is moderate or severe.

In developed countries, about two-thirds of the adult patients who visit the physician because of unexplained stomach problems (dyspepsia) belong to group A, and 2.5–5% belong to group C [83]. In Asian and developing populations, some 20–30% of patients may belong to group A and 20% to group C, on average.

## Diagnosis of *H. pylori* infection

The diagnostic accuracies of both UBT (Urea Breath Test) and stool antigen tests depend on the number of bacteria *(H. pylori)* in the stomach mucosa (colonization). These tests give false-negative results in up to one-half of the patients with AG and acid-free stomach [87]. Moreover, the UBT and stool antigen tests often give false-negative *H. pylori* results in conditions such as bleeding peptic ulcer disease or if the patient is receiving antibiotics or PPIs [88,89].

Serological antibody tests are independent of all the above-mentioned shortcomings, providing that the technical quality and diagnostic validity of the antibodies applied are tested [90,91,92,93,94]. The *H. pylori* tests alone, neither the serological ones, do not give any information of the presence or absence of AG. These tests provide information only of the presence or absence of an ongoing *H. pylori* infection, nothing else.

The Maastricht III Consensus Report accepts validated serological tests as first-line options in the testing *of H. pylori* infection [95].

The “test-and-treat” strategy proposed by the Maastricht Consensus meetings for treatment of dyspepsia in patients under the age of 45 years recommend testing for *H. pylori* alone. This strategy has been criticized in several studies, in particular, from Asia [96,97,98,99]. In these investigations, the *H. pylori* assay alone is not considered reliable enough to exclude the cancer risk in populations in which the stomach cancer (and *H. pylori* gastritis or AG) is frequent also among subjects below the age of 45.

## Diagnosis of AG

Gastroscopy with biopsy microscopy provides an invasive but reliable method (gold standard) for the diagnosis of AG. The biomarker test panel (PGI and PGII, and amidated gastrin-17) is an alternative non-invasive tool to diagnose and screen AG, even at basic health-care centers without endoscopic facilities [29,30,31,32,100,101,102].

### Diagnosis of gastritis and AG with the biomarkers

The sensitivity and specificity of the biomarker test panel in AG were 71–83% and 95–98%, respectively, when a commercial test panel (GastroPanel®, Helsinki, Finland) was validated against the findings in endoscopic biopsy histology in consecutive series of 404 outpatients with dyspepsia and in a population-based sample of approximately 1000 North European subjects undergoing gastroscopy [12,103]. Correspondingly, the sensitivity and specificity of the biomarker test panel to diagnose normal and “healthy” (no *H. pylori* gastritis, no AG) stomach mucosa in the population-based sample of the 1000 subjects were 89% (95% CI 86–92%) and 92% (90–95%), respectively [12].

The PGI test and the ratio of PGI and PGII have been used in the diagnostics of atrophic corpus gastritis worldwide for decades [28,32,104,105,106,107,108,109,110,111,112,113,114,115,116,117].

The knowledge of the plasma level of amidated gastrin-17 together with the PG also enables identification of patients with antral AG and those with extremely extensive AG, i.e., patients with atrophic pangastritis (AG multifocally in the whole stomach). In a case-control study from Japan, the highest cancer risk (risk ratio 25) was observed in patients with low plasma levels of PGI (or ratio PGI/PGII) and with a concomitantly low amidated gastrin-17. All these patients exhibited multifocal AG (AG in both antrum and corpus) in endoscopy and biopsy histology [102].

In *H. pylori* infection, the plasma levels of PGII tend to increase and are often high (>10 μg/l) in the presence of active *Helicobacter* gastritis. Increased plasma levels of PGII are an accessory indicator of an ongoing *H. pylori* infection and are an indirect sign of the “activity” of the *H. pylori* infection [115]. In addition, the plasma levels of PGII may increase in any major gastric irritation, like the NSAID damage, of the stomach mucosa [115].

In assessment of gastric cancer risk by biomarkers in a 10-year follow-up of 5209 asymptomatic middle-aged Japanese, the high plasma levels of *H. pylori* antibodies and the low plasma levels of PGI and/or the ratio of PGI to PGII predicted the cancer risk significantly [116]. The risk ratio reached the level 3.5 (95% CI: 2.0–6.4) when a low PGI (30 μg/l or lower) was used as a criterion and 3.0 (2.5–7.3) when a low ratio (3 or lower) of PGI/PGII ratio was used as a cutoff.

## Value of gastrin-17 as a biomarker of stomach physiology

The plasma levels of amidated G-17 vary extremely rapidly reflecting the normal physiology of the gastrin-acid feedback mechanism in the diurnal control of the acid secretion. The plasma levels of amidated G-17 are sensitive to all physiological stimuli, drugs, and diet. Prolonged use of PPIs raises the plasma levels of gastrin-17 two- to fivefold on average, the rise being dependent on dose and usage of the drugs [118]. The long-lasting use of PPIs and the consequent rise of gastrin-17 will also result in a twofold rise of plasma PGs due to hypertrophy of the oxyntic glands, this hypertrophy, in turn, being likely a consequence of trophic actions of the gastrin to the corpus mucosa [118].

Atrophy of the antral mucosa (loss of antral G cells) leads to a break in feedback control of the acid secretion, i.e., in failure of the synthesis and release of gastrin from the antral mucosa, and results in low fasting and stimulated levels of amidated gastrin-17 in the plasma [103].

In corpus-limited AG, the plasma fasting levels of amidated gastrin-17 are always markedly increased and are several tens of picomoles per liter (normally 2–5 pmol/l) [12,107]. In connection with low plasma PGI and/or low PGI/PGII ratio, the high gastrin-17 in plasma confirms the AG limited in corpus and fundus alone. If the gastrin-17 is not elevated, the AG occurs in both antrum and corpus, i.e., the patient has an extensive and multifocal AG ("panatrophy"; AG of OLGA stage III–IV).

In subjects without *H. pylori* infection or AG (the biomarker panel is normal), the low plasma levels of amidated gastrin-17 (<1–2 pmol/l) are hints of the high output of stomach acid and suggest a high intragastric acidity [34].

In subjects under PPI therapy, low or normal fasting plasma levels of amidated gastrin-17 are a suggestion of antral atrophy. On the other hand, a high plasma level of amidated gastrin-17 in PPI users could be seen as an indicator of normal antral mucosa and suggests that the PPI treatment has lowered the acid output properly.
